# Supplementary material for: Apalutamide Prevents SARS-CoV-2 Infection in Lung Epithelial Cells and in Human Nasal Epithelial Cells
Source: Int J Mol Sci. 2023 Feb 7;24(4):3288. doi: 10.3390/ijms24043288 (PMC9961850; doi:10.3390/ijms24043288)
Supplement: Supplementary file 1 [file ijms-24-03288-s001.zip › ijms-2112049-supplementary.pdf]

Figure S1

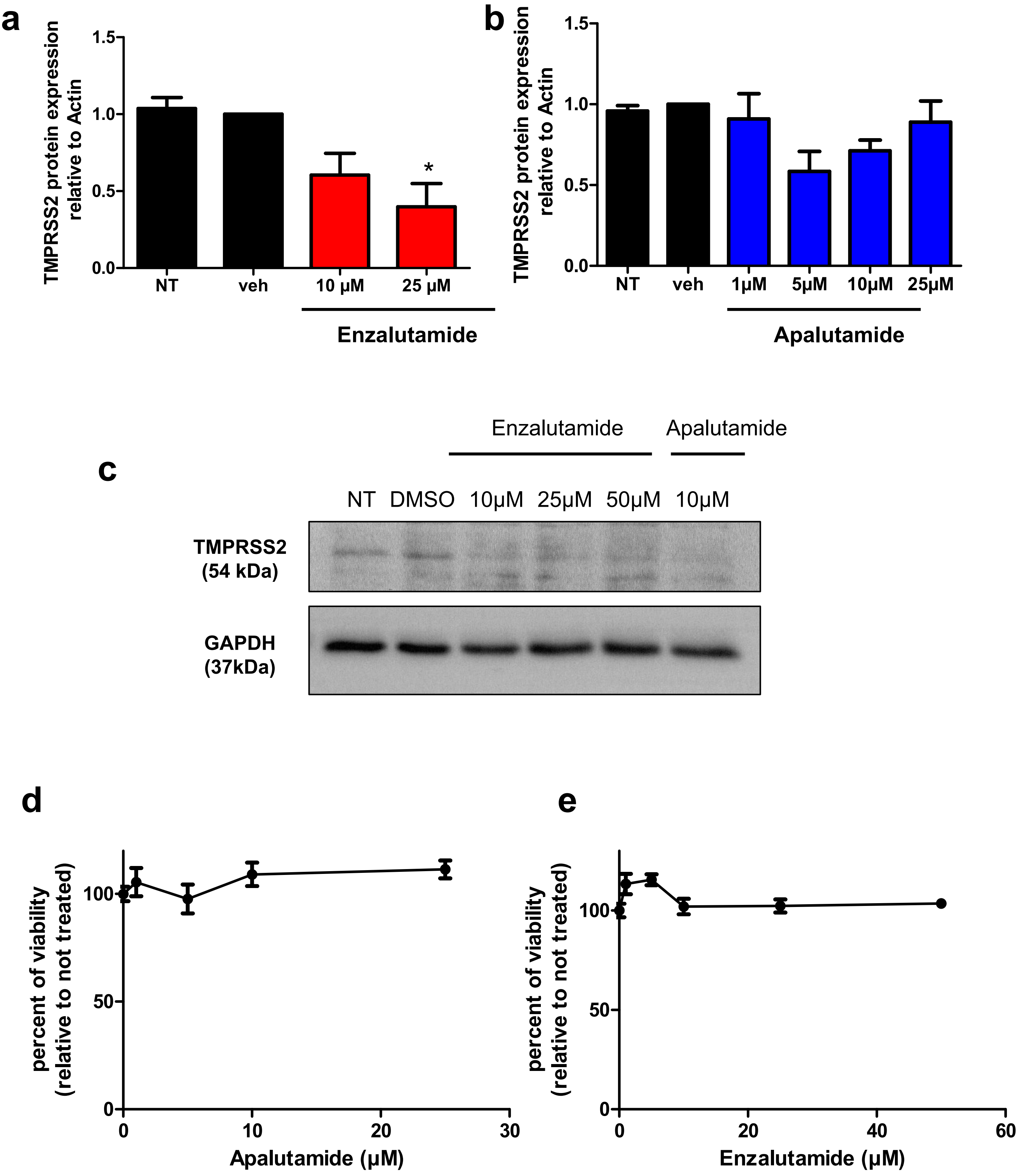
a
TMPRSS2 protein expression relative to Actin
1.5
1.0
0.5
0.0
*
NT
veh
10 µM
25 µM
Enzalutamide
b
TMPRSS2 protein expression relative to Actin
1.5
1.0
0.5
0.0
NT
veh
1µM
5µM
10µM
25µM
Apalutamide
c
Enzalutamide
Apalutamide
NT
DMSO
10µM
25µM
50µM
10µM
TMPRSS2
(54 kDa)
GAPDH
(37kDa)
d
percent of viability (relative to not treated)
100
50
0
0
10
20
30
Apalutamide (µM)
e
percent of viability (relative to not treated)
100
50
0
0
20
40
60
Enzalutamide (µM)

Figure S2

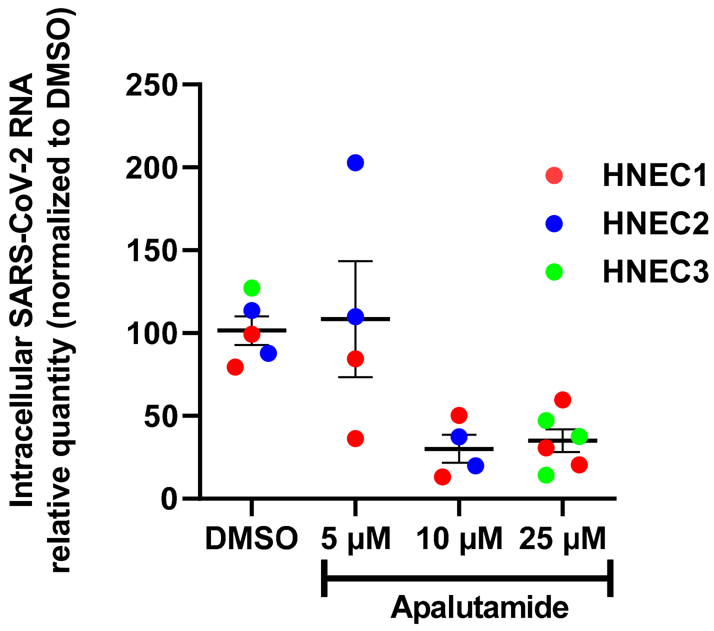

Intracellular SARS-CoV-2 RNA relative quantity (normalized to DMSO)
250
200
150
100
50
0
DMSO
5 µM
10 µM
25 µM
Apalutamide
HNEC1
HNEC2
HNEC3

Figure S3

a

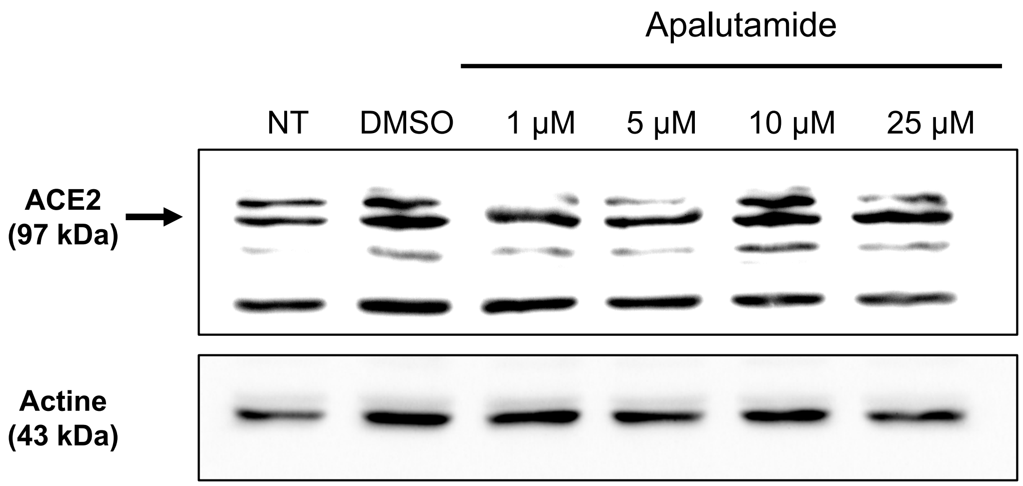
Apalutamide
NT
DMSO
1 µM
5 µM
10 µM
25 µM
ACE2
(97 kDa)
Actine
(43 kDa)

b

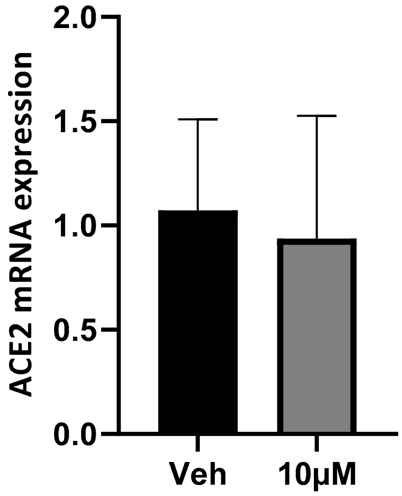
ACE2 mRNA expression
2.0
1.5
1.0
0.5
0.0
Veh
10µM

Figure S1: Effect of AR antagonists on TMPRSS2 expression in lung and prostate cells. (a) Relative quantification of TMPRSS2 to β-actin in response to Enzalutamide, * $p < 0.05$, (b) Relative quantification of TMPRSS2 to β-actin in response to Apalutamide, (c) Western blot analysis of TMPRSS2 and GAPDH in VCaP cells treated by Enzalutamide and Apalutamide (NT: non-treated), (d,e) Viability of Calu-3 cells after 72h treatment by Apalutamide (d) or Enzalutamide (e) measured by MTT.

Figure S2: Effect of Apalutamide on HNECs infection by SARS-CoV-2 viral particles (individual results of Figure 5). Intracellular RNA was extracted from HNECs 72 h post-infection , by SARS-CoV-2 (20 µL of viral inoculum, 2.04×10^5 TCID50/mL) at the apical pole. HNECS were pre-treated for 48 h before infection with DMSO (control) or Apalutamide (5, 10 and 25 µM) at the basal and apical poles, and the drug was maintained for the entire duration of the experiment. SARS-CoV-2 RNA was quantified by RT-qPCR and the results were normalized to 18S rRNA, then to DMSO (control=100%).

Figure S3: ACE2 expression is not regulated by Apalutamide. (a) Western blot analysis of ACE2 and β-actin in Calu-3 cells treated by Apalutamide for 72h (NT: non-treated) (b) Quantification of *ACE2* mRNA in Calu-3 cells treated by 10 µM Apalutamide for 72h as assessed by RT-qPCR. Results were normalized to *RPLP0,* then to Vehicle (DMSO). Relative quantities are expressed as mean$\pm$SEM of three independent experiments.
